# Efficacy and Safety of Sodium-Glucose Cotransporter 2 Inhibitors to Decrease the Risk of Cardiovascular Diseases: A Systematic Review

**DOI:** 10.7759/cureus.44054

**Published:** 2023-08-24

**Authors:** Kiran Prasad Moparthi, Majdah T Al Rushaidi, Meghana Reddy Muddam, Omobolanle A Obajeun, Abdelrahman Abaza, Arturo P Jaramillo, Faten Sid Idris, Humna Anis Shaikh, Ilma Vahora, Tuheen Sankar Nath

**Affiliations:** 1 General Practice, California Institute of Behavioral Neurosciences & Psychology, Fairfield, USA; 2 Psychology, California Institute of Behavioral Neurosciences & Psychology, Fairfield, USA; 3 Pediatrics, California Institute of Behavioral Neurosciences & Psychology, Fairfield, USA; 4 Pathology, California Institute of Behavioral Neurosciences & Psychology, Fairfield, USA; 5 General Surgery, California Institute of Behavioral Neurosciences & Psychology, Fairfield, USA; 6 Surgical Oncology, Tata Medical Centre, Kolkata, IND

**Keywords:** meta-analysis, observational studies, systematic review, sglt2 inhibitors, cardiovascular disorders

## Abstract

Cardiovascular disorders are one of the most frequent causes of death in people throughout the world. These disorders can account for the deaths of 31% of people worldwide. This systematic review examines the effectiveness of sodium-glucose cotransporter 2 (SGLT2) inhibitors in lowering the likelihood of cardiovascular diseases. The study aimed to evaluate various types of research, including randomized controlled trials and observational studies, to analyze how SGLT2 inhibitors impact cardiovascular disorders and establish evidence-based recommendations for clinical practice. The data in this research study were collected from 19 relevant published research articles. The key findings emphasized the potential advantages of SGLT2 inhibitors in reducing major cardiovascular disorders, such as myocardial infarction and stroke. Nonetheless, the study had certain limitations, including reliance on existing literature, exclusion of articles published prior to 2018, and restriction to English-language studies. Despite these limitations, this study contributed significantly to understanding the role of SGLT2 inhibitors in decreasing cardiovascular risk.

## Introduction and background

Cardiovascular disorders are the world's top killer, according to the World Health Organization (WHO). Cardiovascular disorders are one of the most frequent causes of death worldwide [1]. These disorders can account for the deaths of 31% of people all over the world [2]. The decline in the flow of blood to the cardiac muscles is mainly responsible for cardiovascular disorders, which may cause damage or the loss of heart tissue [3]. This is typically caused by plaque formation in the coronary arteries, which can cause blood clots and blockages [4,5]. This can cause chest pain, heart attack, and other symptoms [5,6]. These disorders mainly develop due to an imbalance between the demand and supply of oxygen to the myocardial tissues [7]. Other causes of such disorders include hypertension, partial or total blockage of coronary arteries, coronary artery diseases, blood clots, high blood cholesterol levels, coronary spasms, tobacco smoking, diabetes, high blood triglyceride levels, obesity, alcohol intake, drug abuse, etc. [8]. This blockage can lead to a heart attack and, possibly, death [9,10].

The sodium-glucose cotransporter 2 (SGLT2) inhibitor drugs have emerged as a prospective therapeutic choice for the treatment of cardiovascular disorders [11,12]. Originally developed as SGLT2 inhibitors for the treatment of type 2 diabetes mellitus, these drugs are now being used for cardiovascular disorders as well, owing to their glucose-lowering effects [13-15]. The nephron tubules in the kidneys are the targeted site for these drugs to inhibit glucose reabsorption, increase glucose excretion, and directly lower blood glucose levels [16]. There were several studies available, which support the role of SGLT2 receptor for controlling the level of glucose in the blood [17,18]. The exact mechanisms/way of action of such drugs were not yet fully understood but are believed to involve [19] a combination of factors, such as improved myocardial energetics, reduction in arterial stiffness, and modulation of neurohormonal pathways [20,21].

This systematic review was conducted to study the role of SGLT2 inhibitors in reducing the risk of cardiovascular diseases. The main objectives of this study are to identify and critically evaluate previous research articles to investigate the impact of SGLT2 inhibitors on cardiovascular disorders, evaluate the impact of SGLT2 inhibitors on main cardiovascular disorders such as myocardial infarction, myocardial isthmic, stroke, and cardiovascular death, analyze the functioning ability of SGLT2 inhibitors for the treatment of cardiovascular disorders and providing recommendations for clinical practice, and identify areas for future research in the field of SGLT2 inhibitors [22-24].

The study aims to identify and critically evaluate studies, which contribute to the existing evidence base. By conducting a comprehensive review and analysis of the available literature, this study will synthesize and evaluate the data on the effects of SGLT2 inhibitors on cardiovascular outcomes, adding valuable insights to the current body of knowledge. This knowledge can have significant implications for clinical decision-making and patient management. By investigating the physiological and biochemical pathways through which these medications exert their effects, the study will enhance our understanding of their mechanisms of action. This understanding can uncover the underlying processes involved in cardiovascular risk reduction, leading to insights that may pave more targeted and effective treatment strategies. The generated recommendations can provide guidance to healthcare professionals in making informed decisions regarding the use of SGLT2 inhibitors in managing cardiovascular disorders. Additionally, by identifying areas requiring further investigation, the study will help drive future research efforts, leading to advancements in treatment strategies and improved patient health.

## Review

Materials and methods

Search Sources and Strategy

The Cochrane Library, PubMed, and PubMed Central (PMC) were used as the sources of data for this investigation. Using the Boolean approach, we put together the keywords for a PubMed algorithm.

Eligibility Criteria

In this study, only those research articles were selected, which fulfill the following criteria.

Inclusion criteria: Research articles published from 2018 to 2023, having keywords SGLT2 inhibitors and cardiac disorders, and published in English.

Exclusion criteria: The research articles published before 2018 on SGLT2 inhibitors and cardiovascular diseases were limited, as they were not the main focus, were often not published in English, had very small study populations, and were restricted to specific geographic areas. 

Article Screening and Assessment for Eligibility

The final selection of the research article is based on the inclusion and exclusion criteria. Only those research articles were selected for review, which fulfilled at least 100% of the inclusion and exclusion criteria. Then, the final selected research articles were studied, and the required data were picked for further analysis. Table 1 shows the quality assessment, utilizing the preferred tools, and Table 2 shows the conducted assessment of the quality of observational studies.

**Table 1:** The quality assessment, utilizing the preferred tools: The Joanna Briggs Institute (JBI) and AMSTAR checklist tools.

Types	Tools	Number
Observational Studies	Not Applicable (NEW CASTLE OTTAWA/Joanna Briggs Institute (JBI))	6
Systematic Review	AMSTAR Checklist	1
Meta-Analysis	Preferred Reporting Items for Systematic Reviews and Meta-Analyses (PRISMA) Guidelines	2
Review	N/A	7
Perspective Article	N/A	1
Brief Summary	N/A	1
Journal of the American College of Cardiology (JACC) Review Topic	N/A	1

**Table 2:** The assessment of quality for observational studies is conducted using the Joanna Briggs Institute (JBI) checklist tool.

Authors and year of publication	Were the two groups similar and recruited from the same population?	Were the exposures measured similarly to assign people to both exposed and unexposed groups?	Was the exposure measured in a valid and reliable way?	Were the confounding factors identified?	Were the strategies to deal with confounding factors stated?	Were the groups/participants free of the outcome at the start of the study (or at the time of exposure)?	Were the outcomes measured in a valid and reliable way?	Was the follow-up time reported sufficient to be long enough for outcomes to occur?	Was the follow-up complete, and if not, were the reasons for loss to follow-up described and explored?	Were strategies to address incomplete follow-up utilized?
Andreea et al. 2023 [11]	N/A	Yes	Yes	Yes	Yes	Yes	Yes	N/A	Yes	Yes
Chesterman et al. 2020 [12]	N/A	Yes	Yes	Yes	Yes	Yes	Yes	N/A	Yes	Yes
D'Andrea et al. 2023 [13]	N/A	Yes	Yes	Yes	Yes	Yes	Yes	N/A	Yes	Yes
Han et al. 2021 [15]	N/A	Yes	Yes	Yes	Yes	Yes	Yes	N/A	Yes	Yes
Joshi et al. 2021 [16]	N/A	Yes	Yes	Yes	Yes	Yes	Yes	N/A	Yes	Yes
Kubota et al. 2022 [17]	N/A	Yes	Yes	Yes	Yes	Yes	Yes	N/A	Yes	Yes
Lopaschuk et al. 2020 [18]	N/A	Yes	Yes	Yes	Yes	Yes	Yes	N/A	Yes	Yes
Mascolo et al. 2022 [19]	N/A	Yes	Yes	Yes	Yes	Yes	Yes	N/A	Yes	Yes
Salah et al. 2022 [20]	N/A	Yes	Yes	Yes	Yes	Yes	Yes	N/A	Yes	Yes
Tsai et al. 2022 [21]	N/A	Yes	Yes	Yes	Yes	Yes	Yes	N/A	Yes	Yes
Udell et al. 2022 [22]	N/A	Yes	Yes	Yes	Yes	Yes	Yes	N/A	Yes	Yes
Yang et al. 2020 [23]	N/A	Yes	Yes	Yes	Yes	Yes	Yes	N/A	Yes	Yes
Zhang et al. 2018 [25]	N/A	Yes	Yes	Yes	Yes	Yes	Yes	N/A	Yes	Yes
Pabel et al. 2021 [26]	N/A	Yes	Yes	Yes	Yes	Yes	Yes	N/A	Yes	Yes
Gager et al. 2021 [27]	N/A	Yes	Yes	Yes	Yes	Yes	Yes	N/A	Yes	Yes
Das et al. 2021 [28]	N/A	Yes	Yes	Yes	Yes	Yes	Yes	N/A	Yes	Yes
Dyck et al. 2022 [29]	N/A	Yes	Yes	Yes	Yes	Yes	Yes	N/A	Yes	Yes

Development of Recommendations

The recommendations for clinical practice were developed on the basis of findings of previous articles reviewed in this study. The synthesis involved summarizing the key findings and evaluating their clinical implications. Evidence-based recommendations were formulated to guide healthcare professionals in the use of SGLT2 inhibitors for cardiovascular risk reduction. Additionally, areas for future research were identified, highlighting gaps in the existing knowledge and suggesting potential avenues for further investigation in the field of SGLT2 inhibitors and cardiovascular risk reduction [21].

Results

Search Outcome

**Figure 1:**
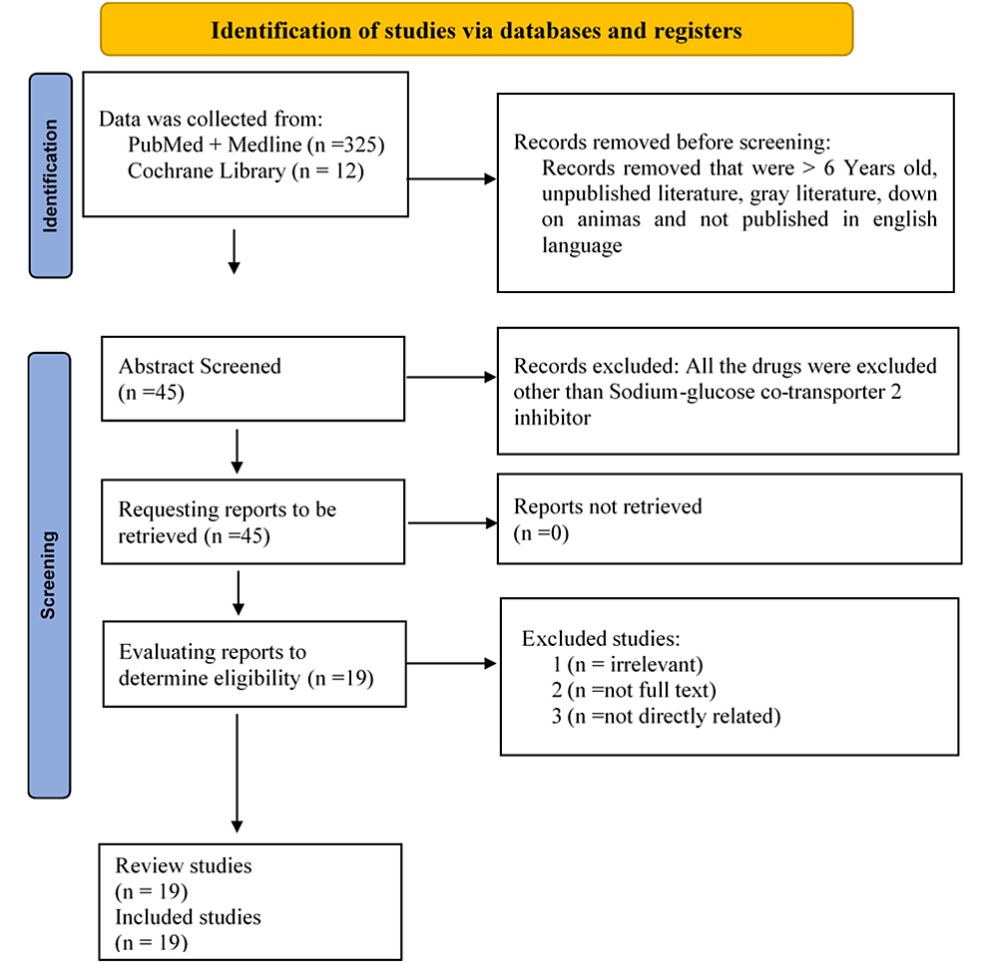
The PRISMA flow diagram illustrates the processes involved in filtering the articles.

In this research, 325 publications in total were examined utilizing MeSH and keywords in various databases. Articles from 2018 to 2023 were included in the search. Included in the search case series reporting on the working ability of SGLT2 inhibitors for the treatment of cardiovascular diseases were among the criteria for inclusion. The study's adult participants had a history of cardiovascular conditions. While the exclusion criteria include any studies that are not specifically about the safety and effectiveness of SGLT2 inhibitors to lower the risk of cardiovascular illnesses, they do include articles published before 2018. The nineteen publications were ultimately chosen for the final evaluation after all irrelevant papers were eliminated from the list. These 19 papers included six observational studies, one systematic review, two meta-analyses, seven review studies, one perspective article, one brief summary, and one JACC evaluation subject. This systematic review and meta-analysis was conducted in accordance with the Preferred Reporting Items for Systematic Reviews and Meta-Analyses (PRISMA) checklist. The PRISMA checklist ensures that the review article meets high standards of transparency and allows readers and researchers to critically evaluate the study's methods and findings [9]. This study aimed to identify and critically assess the (RCTs) and observational studies related to cardiovascular disorders, as well as to assess the impact of SGLT2 inhibitors on major cardiovascular disorders. The article filtering process is shown in the PRISMA flow diagram in Figure 1 [22].

Actual Results

Table 3 summarizes the findings of each paper reviewed in this study. The table presents a collection of studies conducted by different authors in various years, each addressing different aspects of SGLT2 inhibitors [23]. These studies contribute to our understanding of different treatment approaches and factors influencing cardiovascular diseases. A total of 325 research articles were reviewed, out of which only 19 most relevant were included in this review, as shown in Table 3.

**Table 3:** Sodium-glucose cotransporter 2 (SGLT2) inhibitors' ability to effectively and safely reduce the risk of cardiovascular illnesses.

Authors and year of publication	Purpose of the study	Number of patients/procedures used	Type of study	Conclusion
Andreea et al. 2023 [11]	Unforeseen advantages or adverse effects of sodium-glucose cotransporter 2 (SGLT2) inhibitors	N/A	Review	The potential harms and benefits of SGLT2 inhibitors as a therapeutic option, indicating the importance of conducting a balanced assessment to determine their overall impact on patient outcomes
Chesterman et al. 2020 [12]	The pros and cons of sodium-glucose cotransporter 2 (SGLT2) inhibitors	N/A	Review	This study highlights the need for careful consideration of both the potential risks and advantages associated with SGLT2 inhibitors to optimize patient outcomes and ensure their safe and effective utilization in clinical practice
D’Andrea et al. 2023 [13]	SGLT2 inhibitors vs DPP-4 inhibitors	N/A	Review	This study highlights the importance of considering individual patient characteristics, including baseline HbA1c levels, when selecting between SGLT2 inhibitors and DPP-4 inhibitors for optimal outcomes in type 2 diabetes management
Goit et al. 2019 [14]	Pharmacological management of patients with type 2 diabetes and cardiovascular disease: a review	N/A	Review	The rising prevalence of type 2 diabetes and its associated cardiovascular (CV) complications pose a significant economic burden. To address this, treatment strategies need to extend beyond glucose control, focusing on event-driven approaches. Fortunately, recent years have witnessed the efficacy of novel anti-diabetic agents in reducing major CV outcomes, heart failure, and end-stage renal disease. This review article presents consensus guidelines for treating type 2 diabetes patients with different categories of CV disease, offering updated information on HbA1c targets and drug selection
Han et al. 2021 [15]	Sodium-glucose cotransporter 2 (SGLT2) inhibitors with dipeptidyl peptidase-4 (DPP-4) inhibitors in elderly individuals with type 2 diabetes	N/A	Observational Study	This study provides valuable evidence for clinical decision-making by demonstrating the effectiveness and safety of sodium-glucose cotransporter-2 (SGLT2) inhibitors compared to dipeptidyl peptidase-4 (DPP-4) inhibitors in older adults with type 2 diabetes. The findings support SGLT2 inhibitors as a favorable treatment option, offering improved glycemic control and potentially reduced risk of adverse cardiovascular events when compared to DPP-4 inhibitors in this population
Joshi et al. 2021 [16]	Mechanisms of action of sodium-glucose cotransporter 2 (SGLT2) inhibitor therapy in heart failure	N/A	Review	The mechanisms of action of sodium-glucose cotransporter 2 (SGLT2) inhibitors in heart failure are explored, revealing their potential therapeutic benefits in enhancing cardiac function. The findings underscore the significance of SGLT2 inhibitors as a promising treatment option for heart failure, as they operate through novel mechanisms that extend beyond glycemic control and have the potential to positively influence cardiovascular outcomes
Kubota et al. 2022 [17]	Clinical advantages of sodium-glucose cotransporter 2 inhibitors and the cardiovascular effects caused by these drugs	N/A	Review	This article highlights the clinical benefits of sodium-glucose cotransporter 2 (SGLT2) inhibitors and explores the underlying mechanisms responsible for their cardiovascular effects. The findings underscore the significant cardiovascular benefits of SGLT2 inhibitors, emphasizing their potential as an effective therapeutic option and contributing to improved patient outcomes in cardiovascular disease management
Lopaschuk et al. 2020 [18]	Mechanisms of sodium glucose cotransporter 2 (SGLT2) inhibitors' cardiovascular advantages	N/A	N/A	This article highlights the potential of SGLT2 inhibitors to improve cardiovascular outcomes by targeting multiple pathways, offering valuable knowledge for advancing clinical practice and further research in this field
Mascolo et al. 2022 [19]	Sodium-glucose cotransporter 2 (SGLT2) inhibitor safety profile	N/A	Brief summary	This article provides a concise overview of the safety profile of sodium-glucose cotransporter 2 (SGLT2) inhibitors, highlighting their potential as a safe treatment option in cardiovascular medicine
Salah et al. 2022 [20]	Sodium-glucose cotransporter 2 and acute heart failure who have type 2 diabetes or not	N/A	Systematic Review + Meta-analysis	The SGLT2 inhibitors show efficacy and safety in patients with acute heart failure, with and without type 2 diabetes
Tsai et al. 2022 [21]	Sodium-glucose cotransporter-2 inhibitors' and cardiovascular and renal systems in people without diabetes	N/A	Systematic Review + Meta-analysis	The SGLT2 inhibitors show cardiovascular and renal efficacy and safety in patients without diabetes
Udell et al. 2022 [22]	For acute myocardial infarction, sodium glucose cotransporter-2 inhibition is used	N/A	Review	This article highlights the potential benefits of sodium-glucose cotransporter-2 (SGLT2) inhibition as a therapeutic strategy for acute myocardial infarction, suggesting its promising role in improving outcomes for patients
Yang et al. 2020 [23]	Inhibitors of sodium-glucose cotransporter-2 and their effects on the heart	N/A	Review	This article provides an overview of the cardiovascular effects and underlying mechanisms of sodium-glucose cotransporter-2 (SGLT2) inhibitors, shedding light on their potential therapeutic impact in managing chronic diseases
Yang et al. 2019 [24]	Sodium-glucose cotransporter 2 inhibitors' effectiveness and security in treating type 2 diabetes in East Asians	N/A	Meta-analysis	The SGLT2 inhibitors show efficacy and safety in East Asians with type 2 diabetes
Zhang et al. 2018 [25]	Inhibitors of sodium-glucose cotransporter 2's cardiovascular safety, long-term no cardiovascular safety, and effectiveness in people with type 2 diabetes	N/A	Systematic Review + Meta-analysis	The SGLT2 inhibitors show cardiovascular safety, long-term non-cardiovascular safety, and efficacy in patients with type 2 diabetes mellitus
Pabel et al. 2021 [26]	Has the riddle around SGLT2 inhibitors and their method of action in heart failure been solved?	N/A	Review	This article explores the mode of action of SGLT2 inhibitors in heart failure and discusses the potential mechanisms behind their beneficial effects. It suggests that while significant progress has been made in understanding their actions, further research is still needed to fully unravel the complexities of their therapeutic impact
Gager et al. 2021 [27]	A meta-analysis of cardiovascular outcomes in individuals treated for heart failure with SGLT2 inhibitors	N/A	Meta-analysis	The SGLT2 inhibitors show favorable cardiovascular outcomes in patients with heart failure
Das et al. 2021 [28]	Heart failure with a lower ejection fraction and SGLT2 inhibitors	N/A	Review	This article focuses on the use of SGLT2 inhibitors in heart failure patients with reduced ejection fraction, highlighting their potential benefits in improving clinical outcomes and reducing mortality. Further studies are warranted to explore their long-term efficacy and safety in this patient population
Dyck et al. 2022 [29]	Evidence for possible off-target effects in the cardiac mechanisms underlying the therapeutic benefits of SGLT2 inhibitors in heart failure	N/A	Review	This article provides insights into the cardiac mechanisms underlying the beneficial effects of SGLT2 inhibitors in heart failure, suggesting potential off-target effects beyond their primary mode of action. These findings emphasize the need for further investigation to fully comprehend the multifaceted mechanisms contributing to the therapeutic benefits of SGLT2 inhibitors in heart failure

Discussion

SGLT2 Inhibitors and Their Role in Reducing Risk of Cardiovascular Diseases

SGLT2 inhibitors are a group of medications commonly used in the management of type 2 diabetes [24]. These drugs work by inhibiting the SGLT2 protein, which is responsible for reabsorbing glucose in the kidneys and returning it to the bloodstream [25,26]. By blocking this protein, SGLT2 inhibitors promote the excretion of excess glucose through urine, leading to lowered blood sugar levels [27]. Beyond their primary role in diabetes management, SGLT2 inhibitors have emerged as potential agents for reducing the risk of cardiovascular diseases. Multiple clinical trials and observational studies have demonstrated that these medications offer additional cardiovascular benefits beyond glucose control [28,29]. They have been shown to decrease the risk of major adverse cardiovascular events, including heart attacks, strokes, and cardiovascular death [15]. The mechanisms underlying the cardiovascular benefits of SGLT2 inhibitors are still under investigation [30]. It is believed that these drugs may exert their positive effects through various pathways, such as reducing blood pressure, promoting weight loss, improving arterial function, and reducing inflammation [31].

Comparison with the Existing Literature

The present study's comparison with existing literature revealed several key findings and contributions [14,24]. This study developed and evaluated the available evidence for the impacts of SGLT2 inhibitors in lowering cardiovascular diseases, providing a comprehensive overview of the current knowledge in this field [32-34]. The inclusion of relevant RCTs and observational studies added to the existing evidence base, while the evaluation of major cardiovascular disorders, such as myocardial infarction, myocardial ischemia, stroke, and cardiovascular death provided a deeper understanding of the specific effects of SGLT2 inhibitors on cardiovascular outcomes [35,36]. The study's analysis working ability of SGLT2 inhibitors contributed to our understanding of how these medications treat cardiovascular disorders [37]. Evidence-based recommendations for clinical practice and the identification of areas for future research further aligned this study with the existing literature, facilitating the translation of findings into clinical decision-making and guiding future research efforts [38].

The study conducted by Andreea et al. [11] revealed that the SGLT2 inhibitors offer unexpected benefits beyond their glucose-lowering effects. The findings of these drugs indicate that these medications provide significant cardiovascular benefits, including reducing the risk of major adverse cardiovascular events, such as heart attacks, strokes, cardiovascular death, etc. [39,40]. These drugs also help lower blood pressure, promote weight loss, improve arterial function, and reduce inflammation. Another conducted by Chesterman et al. [12] supports the findings of Andreea et al. [11] and reveals that SGLT2 inhibitors have emerged as a valuable therapeutic option for the treatment of cardiovascular disorders [41]. The clinical trials and real-world evidence show that these medications can reduce the risk of major adverse cardiovascular events, including heart attacks, strokes, and cardiovascular death. Another study conducted by D’Andrea et al. [13] containing a large sample size of 144,614 adults with type 2 diabetes (mean age 62 years, 54% male), initiating treatment with sodium-glucose cotransporter 2 (SGLT2) inhibitors showed promising results in decreasing the risk of cardiovascular diseases [41,42]. The findings of this study highlight the potential of SGLT2 inhibitors in reducing cardiovascular risks in patients with type 2 diabetes.

The findings of the study conducted by Han et al. [15] reported that SGLT2 inhibitors play a crucial role in decreasing the risk of cardiovascular diseases. Further, SGLT2 inhibitors are involved in reducing the risks of diabetic ketoacidosis, bone fracture, and severe hypoglycemia. This suggests that SGLT2 inhibitors not only provide cardiovascular disease protection but are also safe for older adults with type 2 diabetes. Joshi et al. [16] reported similar findings and concluded that SGLT2 inhibitors are the most advanced and effective therapeutic option for patients with type 2 diabetes mellitus (T2DM) and cardiovascular diseases due to their ability to improve blood glucose control [43]. However, its exact working mechanisms are not yet fully understood.

A recent review study conducted by Kubota et al. [17] concluded that the SGLT2 inhibitors exhibit antidiabetic effects and play a crucial role in reducing cardiovascular events in patients with T2DM. The findings of this study show that the SGLT2 inhibitors improve cardiovascular and renal outcomes, leading to reduced rehospitalization rates among patients with heart failure, regardless of their diabetes status. The findings of another review study reported by Lopaschuk et al. [18] support the findings of Kubota et al. [17]. This study demonstrated the remarkable cardiovascular benefits of SGLT2 inhibitors. The findings of this study revealed that the SGLT2 inhibitors involved in diuresis/natriuresis, blood pressure reduction, improved cardiac energy metabolism, inflammation reduction, inhibition of the sympathetic nervous system, prevention of adverse cardiac remodeling, protection against ischemia/reperfusion injury, inhibition of the Na+/H+ exchanger and SGLT1, reduction in hyperuricemia, increased autophagy and lysosomal degradation, decreased epicardial fat mass, elevated erythropoietin levels, augmented pro-vascular progenitor cell circulation, decreased oxidative stress, and improved vascular function.

A study conducted by Mascolo et al. [19] reported that SGLT2 inhibitors are a new therapeutic class of oral agents. These inhibitors are involved in blocking SGLT2 in the renal tubules and preventing the reabsorption of glucose and sodium. This leads to increased glucose excretion in the urine and reduced blood glucose levels, which directly or indirectly reduces the chances of cardiovascular disorders. Salah et al. [20] conducted a systematic review and meta-analysis that also supports the findings of Mascolo et al. [19] and revealed that SGLT2 inhibitors are involved in reducing the risk of rehospitalization for heart failure. Similar findings were reported by Udell et al. [22] who concluded that SGLT2 inhibitors play an important role in improving cardiorenal outcomes in patients with T2DM, chronic kidney disease, and chronic heart failure [44].

Yang et al. [23] conducted a study and revealed that SGLT2 inhibitors are a new and effective class of drugs for diabetes treatment and exhibit significant hypoglycemic effects. This study also concluded that GLT2 inhibitors are also involved in the reduction of atherosclerosis, hospitalization for heart failure, and cardiovascular death. A review study reported by Yang et al. [24] concluded that SGLT2 inhibitors have a positive impact on East Asian patients with T2DM and cardiovascular diseases in terms of efficacy and safety. Zhang et al. [25] also supported the findings of Yang et al. [23] and concluded that the SGLT2 inhibitors have a positive impact on reducing the risk of cardiovascular diseases. The findings of this study provide robust evidence supporting the cardiovascular and long-term noncardiovascular safety and efficacy of SGLT2 inhibitors in the treatment of patients. The findings of Gager et al. [27] concluded that SGLT2 inhibitors play a significant role in decreasing the risk of cardiovascular diseases, particularly in patients with heart failure (HF) [45]. This study also revealed that SGLT2 inhibitors demonstrated a 27% relative risk reduction in the composite outcome of hospitalization for HF or cardiovascular (CV) mortality, a 32% risk reduction in hospitalization for HF, an 18% risk reduction in CV mortality, and a 17% risk reduction in all-cause mortality.

Summary of the Findings

The aim of this study is to assess the impact of SGLT2 inhibitors on minimizing the risk of cardiovascular diseases. The analysis included a range of RCTs and observational studies, which provided valuable insights into the effects of SGLT2 inhibitors on major CVDs, such as myocardial infarction, myocardial ischemia, stroke, and cardiovascular death. The findings of this study align with previous research, indicating that SGLT2 inhibitors offer unexpected benefits beyond glucose-lowering effects. These medications have been shown to significantly reduce the risk of major adverse cardiovascular events and improve outcomes in patients with heart failure. Evidence-based recommendations provided in this study contribute to clinical practice and guide future research efforts. Other studies also support these findings, highlighting the ability of SGLT2 inhibitors to lower CVD risks, reduce blood pressure, promote weight loss, improve arterial function, and decrease inflammation [46]. They are considered a valuable therapeutic option for patients with T2DM and cardiovascular diseases. The mechanisms of action for SGLT2 inhibitors are not yet fully understood, but they exhibit antidiabetic effects and have a positive impact on cardiovascular and renal outcomes. These medications have demonstrated multiple benefits, including diuresis/natriuresis, blood pressure reduction, improved cardiac energy metabolism, inflammation reduction, and protection against adverse cardiac remodeling [47]. They also contribute to decreased oxidative stress, improved vascular function, and reduced rehospitalization rates for heart failure. The safety and efficacy of SGLT2 inhibitors have been well-documented, providing robust evidence for their use in the treatment of patients with T2DM and cardiovascular disorders.

Limitations of the Study

The limitations of this study include the following. This study may be subjected to publication bias as it only includes articles published in English and within a specific timeframe (2018-2023). Relevant studies published in other languages or before 2018 might have been missed, potentially leading to a biased representation of the available literature. The inclusion and exclusion criteria used to select articles for the review may introduce selection bias. By excluding studies with small populations or restricted to specific areas, certain valuable data might have been excluded, potentially impacting the generalizability of the study's findings [48]. The quality and reliability of the included studies may vary, as indicated by the assessment using different tools. The heterogeneity in study design, methodology, and reporting quality among the selected articles could affect the overall strength of the evidence and the validity of the conclusions drawn. The findings of the study may not be generalizable to broader populations or different settings, as the majority of the included studies might have focused on specific patient populations or regions [49]. This limitation should be considered when applying the study's recommendations to clinical practice in diverse contexts. The review may be limited by the availability of long-term data on the safety and efficacy of SGLT2 inhibitors [50]. As the study focuses on articles published up to 2023, the long-term effects and outcomes beyond that time frame may not be adequately captured. The included studies in the systematic review may have used different study designs, such as RCTs and observational studies, which can introduce inherent limitations and biases [51]. Combining data from diverse study designs may present challenges in interpreting and synthesizing the results.

Future Research Directions

This study recommended the following on the basis of the findings of this systematic review; healthcare professionals should consider incorporating SGLT2 inhibitors as part of the treatment strategy for patients with cardiovascular diseases, given the potential benefits observed in reducing the risk of major cardiovascular disorders [52,53]. Efforts should be made to enhance patient education and awareness about the potential benefits and risks of SGLT2 inhibitors, facilitating shared decision-making between healthcare providers and patients and future research should focus on addressing the limitations of existing studies, including the need for larger-scale randomized controlled trials and studies conducted in non-English language settings, to strengthen the evidence base and provide more robust recommendations for clinical practice.

## Conclusions

This systematic review highlights the key findings regarding the impact of SGLT2 inhibitors in decreasing the probability of cardiovascular diseases. The review identified a significant body of evidence supporting the use of SGLT2 inhibitors for cardiovascular risk reduction, particularly in terms of reducing the incidence of myocardial infarction, stroke, and cardiovascular death. This study emphasizes the clinical and practical implications of incorporating SGLT2 inhibitors as part of the treatment strategy for patients with cardiovascular diseases. Healthcare professionals should consider the potential benefits of SGLT2 inhibitors and stay updated on the evolving evidence to make informed decisions in managing patients with cardiovascular disorders. Furthermore, patient education and shared decision-making should be encouraged to ensure the appropriate use of SGLT2 inhibitors in clinical practice.

## References

[1] Balakumar P, Maung-U K, and Jagadeesh G (2016). Prevalence and prevention of cardiovascular disease and diabetes mellitus. Pharmacol Res.

[2] Mc Namara K, Alzubaidi H, Jackson JK (2019). Cardiovascular disease as a leading cause of death: how are pharmacists getting involved?. Integr Pharm Res Pract.

[3] Khan MA, Hashim MJ, Mustafa H (2020). Global epidemiology of ischemic heart disease: results from the global burden of disease study. Cureus.

[4] Nowbar AN, Gitto M, Howard JP, Francis DP, Al-Lamee R (2019). Mortality from ischemic heart disease. Circ Cardiovasc Qual Outcomes.

[5] Glovaci D, Fan W, Wong ND (2019). Epidemiology of diabetes mellitus and cardiovascular disease. Curr Cardiol Rep.

[6] Mattioli AV, Sciomer S, Cocchi C, Maffei S, Gallina S (2020). Quarantine during COVID-19 outbreak: changes in diet and physical activity increase the risk of cardiovascular disease. Nutr Metab Cardiovasc Dis.

[7] Roth GA, Johnson C, Abajobir A (2017). Global, regional, and national burden of cardiovascular diseases for 10 causes, 1990 to 2015. J Am Coll Cardiol.

[8] Ritchie H, Spooner F, and Roser M (2023). Causes of death. https://ourworldindata.org/causes-of-death.

[9] Page MJ, McKenzie JE, Bossuyt PM (2021). The PRISMA 2020 statement: an updated guideline for reporting systematic reviews. BMJ.

[10] Timmis A, Townsend N, Gale C (2018). European society of cardiology: cardiovascular disease statistics 2017. Eur Heart J.

[11] Andreea MM, Surabhi S, Razvan-Ionut P, Lucia C, Camelia N, Emil T, Tiberiu NI (2023). Sodium-glucose cotransporter 2 (SGLT2) inhibitors: harms or unexpected benefits?. Medicina (Kaunas).

[12] Chesterman T, Thynne TR (2020). Harms and benefits of sodium-glucose co-transporter 2 inhibitors. Aust Prescr.

[13] D'Andrea E, Wexler DJ, Kim SC, Paik JM, Alt E, Patorno E (2023). Comparing effectiveness and safety of SGLT2 inhibitors vs DPP-4 inhibitors in patients with type 2 diabetes and varying baseline HbA1c levels. JAMA Intern Med.

[14] Goit LN, Shaning Y (2019). Pharmacological management of patients with type 2 diabetes and cardiovascular disease. International Journal of Science Inventions Today.

[15] Han SJ, Ha KH, Lee N, Kim DJ (2021). Effectiveness and safety of sodium-glucose co-transporter-2 inhibitors compared with dipeptidyl peptidase-4 inhibitors in older adults with type 2 diabetes: a nationwide population-based study. Diabetes Obes Metab.

[16] Joshi SS, Singh T, Newby DE, Singh J (2021). Sodium-glucose co-transporter 2 inhibitor therapy: mechanisms of action in heart failure. Heart.

[17] Kubota Y, Shimizu W (2022). Clinical benefits of sodium-glucose cotransporter 2 inhibitors and the mechanisms underlying their cardiovascular effects. JACC: Asia.

[18] Lopaschuk GD, Verma S (2020). Mechanisms of cardiovascular benefits of sodium glucose co-transporter 2 (SGLT2) inhibitors: a state-of-the-art review. JACC Basic Transl Sci.

[19] Mascolo A, Di Napoli R, Balzano N (2022). Safety profile of sodium glucose co-transporter 2 (SGLT2) inhibitors: a brief summary. Front Cardiovasc Med.

[20] Salah HM, Al'Aref SJ, Khan MS (2022). Efficacy and safety of sodium-glucose cotransporter 2 inhibitors initiation in patients with acute heart failure, with and without type 2 diabetes: a systematic review and meta-analysis. Cardiovasc Diabetol.

[21] Tsai WC, Hsu SP, Chiu YL (2022). Cardiovascular and renal efficacy and safety of sodium-glucose cotransporter-2 inhibitors in patients without diabetes: a systematic review and meta-analysis of randomised placebo-controlled trials. BMJ Open.

[22] Udell JA, Jones WS, Petrie MC (2022). Sodium glucose cotransporter-2 inhibition for acute myocardial infarction: JACC Review topic of the week. J Am Coll Cardiol.

[23] Yang F, Meng R, Zhu DL (2020). Cardiovascular effects and mechanisms of sodium-glucose cotransporter-2 inhibitors. Chronic Dis Transl Med.

[24] Yang L, Zhang L, He H, Zhang M, An Z (2019). Efficacy and safety of sodium-glucose cotransporter 2 inhibitors in east Asians with type 2 diabetes: a systematic review and meta-analysis. Diabetes Ther.

[25] Zhang XL, Zhu QQ, Chen YH, Li XL, Chen F, Huang JA, Xu B (2018). Cardiovascular safety, long-term noncardiovascular safety, and efficacy of sodium-glucose cotransporter 2 inhibitors in patients with type 2 diabetes mellitus: a systemic review and meta-analysis with trial sequential analysis. J Am Heart Assoc.

[26] Pabel S, Hamdani N, Luedde M (2021). SGLT2 inhibitors and their mode of action in heart failure—has the mystery been unravelled?. Curr Heart Fail Rep.

[27] Gager GM, Gelbenegger G, Jilma B (2021). Cardiovascular outcome in patients treated with SGLT2 inhibitors for heart failure: a meta-analysis. Front Cardiovasc Med.

[28] Aziri B, Begic E, Jankovic S (2023). Systematic review of sodium‐glucose cotransporter 2 inhibitors: a hopeful prospect in tackling heart failure‐related events. ESC Heart Fail.

[29] Dyck JR, Sossalla S, Hamdani N, Coronel R, Weber NC, Light PE, Zuurbier CJ (2022). Cardiac mechanisms of the beneficial effects of SGLT2 inhibitors in heart failure: evidence for potential off-target effects. J Mol Cell Cardiol.

[30] Bichenapally S, Khachatryan V, Muazzam A (2022). Risk of liver fibrosis in methotrexate-treated patients: a systematic review. Cureus.

[31] Nakano H, Minami I, Braas D (2017). Glucose inhibits cardiac muscle maturation through nucleotide biosynthesis. Elife.

[32] Ashtari K, Nazari H, Ko H (2019). Electrically conductive nanomaterials for cardiac tissue engineering. Adv Drug Deliv Rev.

[33] Waters R, Alam P, Pacelli S, Chakravarti AR, Ahmed RP, Paul A (2018). Stem cell-inspired secretome-rich injectable hydrogel to repair injured cardiac tissue. Acta Biomater.

[34] Kato ET, Kimura T (2020). Sodium-glucose co-transporters-2 inhibitors and heart failure: state of the art review and future potentials. Int J Heart Fail.

[35] Vaduganathan M, Januzzi JL Jr (2019). Preventing and treating heart failure with sodium-glucose co-transporter 2 inhibitors. Am J Cardiol.

[36] Usman MS, Siddiqi TJ, Memon MM (2018). Sodium-glucose co-transporter 2 inhibitors and cardiovascular outcomes: a systematic review and meta-analysis. Eur J Prev Cardiol.

[37] Rajeev SP, Cuthbertson DJ, Wilding JP (2016). Energy balance and metabolic changes with sodium-glucose co-transporter 2 inhibition. Diabetes Obes Metab.

[38] Sarzani R, Giulietti F, Di Pentima C, Spannella F (2020). Sodium-glucose co-transporter-2 inhibitors: peculiar "hybrid" diuretics that protect from target organ damage and cardiovascular events. Nutr Metab Cardiovasc Dis.

[39] Tentolouris A, Vlachakis P, Tzeravini E, Eleftheriadou I, Tentolouris N (2019). SGLT2 inhibitors: a review of their antidiabetic and cardioprotective effects. Int J Environ Res Public Health.

[40] Hajar R (2016). Framingham contribution to cardiovascular disease. Heart Views.

[41] Stewart RA, Wallentin L, Benatar J (2016). Dietary patterns and the risk of major adverse cardiovascular events in a global study of high-risk patients with stable coronary heart disease. Eur Heart J.

[42] Ettehad D, Emdin CA, Kiran A (2016). Blood pressure lowering for prevention of cardiovascular disease and death: a systematic review and meta-analysis. Lancet.

[43] O'Meara E, McDonald M, Chan M (2020). CCS/CHFS heart failure guidelines: clinical trial update on functional mitral regurgitation, SGLT2 inhibitors, ARNI in HFpEF, and tafamidis in amyloidosis. Can J Cardiol.

[44] Ali A, Bain S, Hicks D (2019). SGLT2 inhibitors: cardiovascular benefits beyond HbA1c—translating evidence into practice. Diabetes Ther.

[45] Auerbach JM, and Khera M (2020). Hypogonadism management and cardiovascular health. Postgrad Med.

[46] Aung T (2018). The role of omega-3 fatty acids and aspirin in the prevention of cardiovascular disease in diabetes and biochemical effectiveness of omega-3 fatty acids and aspirin in the ASCEND trial (Doctoral dissertation). Queen Mary Univ Lond.

[47] Medina-Remón A, Kirwan R, Lamuela-Raventós RM, Estruch R (2018). Dietary patterns and the risk of obesity, type 2 diabetes mellitus, cardiovascular diseases, asthma, and neurodegenerative diseases. Crit Rev Food Sci Nutr.

[48] Tremblay LJ (2017). Nurse practitioner impact on quantitative patient outcomes in four healthcare settings' system context: a systematic review and meta-analysis. Univ Saskatchewan.

[49] Ramos R, Comas-Cufí M, Martí-Lluch R (2018). Statins for primary prevention of cardiovascular events and mortality in the old and very old population. PubMed.

[50] Schofield P, Dunham M, Martin D (2019). National guidelines for the management of pain in older adults. Edinb Napier Res Repos.

[51] Varghese TP, Kumar AV (2019). Predisposing risk factors of acute coronary syndrome (ACS): a mini review. J Pharm Sci Res.

[52] Tajfard M, Tavakoly Sany SB, Avan A (2019). Relationship between serum high sensitivity C‐reactive protein with angiographic severity of coronary artery disease and traditional cardiovascular risk factors. J Cell Physiol.

[53] Nakagawa Y, Kuwahara K (2020). Sodium-glucose cotransporter-2 inhibitors are potential therapeutic agents for treatment of non-diabetic heart failure patients. J Cardiol.

